# Biochemical and Functional Characterization of Mouse Mammary Tumor Virus Full-Length Pr77^Gag^ Expressed in Prokaryotic and Eukaryotic Cells

**DOI:** 10.3390/v10060334

**Published:** 2018-06-18

**Authors:** Akhil Chameettachal, Vineeta Narayana Pillai, Lizna Mohamed Ali, Fathima Nuzra Nagoor Pitchai, Mustafa Taleb Ardah, Farah Mustafa, Roland Marquet, Tahir Aziz Rizvi

**Affiliations:** 1 Department of Microbiology & Immunology, College of Medicine and Health Sciences (CMHS), United Arab Emirates University (UAEU), Al Ain 20000, United Arab Emirates (UAE); akhilc341@uaeu.ac.ae (A.C.); vineeta.pillai@uaeu.ac.ae (V.N.P.); lizna@uaeu.ac.ae (L.M.A.); f_nuzra@uaeu.ac.ae (F.N.N.P.); 2 Department of Biochemistry, College of Medicine and Health Sciences (CMHS), United Arab Emirates University (UAEU), Al Ain 20000, United Arab Emirates (UAE); mustafa_ardah@uaeu.ac.ae (M.T.A.); fmustafa@uaeu.ac.ae (F.M.); 3 Centre National de la Recherche Scientifique (CNRS), Architecture et Réactivité de l’ARN, UPR 9002, Université de Strasbourg, 67084 Strasbourg, France; r.marquet@ibmc-cnrs.unistra.fr

**Keywords:** retrovirus, mouse mammary tumor virus (MMTV), RNA–protein interaction, protein assembly, protein expression, protein purification, RNA packaging, RNA–Gag interactions, Pr77^Gag^

## Abstract

The mouse mammary tumor virus (MMTV) Pr77^Gag^ polypeptide is an essential retroviral structural protein without which infectious viral particles cannot be formed. This process requires specific recognition and packaging of dimerized genomic RNA (gRNA) by Gag during virus assembly. Most of the previous work on retroviral assembly has used either the nucleocapsid portion of Gag, or other truncated Gag derivatives—not the natural substrate for virus assembly. In order to understand the molecular mechanism of MMTV gRNA packaging process, we expressed and purified full-length recombinant Pr77^Gag^-His_6_-tag fusion protein from soluble fractions of bacterial cultures. We show that the purified Pr77^Gag^-His_6_-tag protein retained the ability to assemble virus-like particles (VLPs) in vitro with morphologically similar immature intracellular particles. The recombinant proteins (with and without His_6_-tag) could both be expressed in prokaryotic and eukaryotic cells and had the ability to form VLPs in vivo. Most importantly, the recombinant Pr77^Gag^-His_6_-tag fusion proteins capable of making VLPs in eukaryotic cells were competent for packaging sub-genomic MMTV RNAs. The successful expression and purification of a biologically active, full-length MMTV Pr77^Gag^ should lay down the foundation towards performing RNA–protein interaction(s), especially for structure-function studies and towards understanding molecular intricacies during MMTV gRNA packaging and assembly processes.

## 1. Introduction

The mouse mammary tumor virus (MMTV) is an oncogenic retrovirus that causes both breast cancer and lymphoma/leukemia in mice. It can be transmitted to the progeny exogenously through the breast milk or vertically through the germline as endogenous viruses [1,2,3,4]. This makes MMTV a suitable model for studying the mechanism of oncogenesis and the genetics involved in the development of mammary tumors [5,6]. Furthermore, it has unique genetic properties that make MMTV a desirable vector system for delivering therapeutic genes in human gene transfer studies. The advantages of MMTV-based vectors include: (i) being phylogenetically distinct from human and primate retroviruses, reducing the chances of recombination with endogenous human viruses; (ii) an ability to transduce non-dividing cells, the main target cells of human gene therapy [7]; (iii) containing multiple promoters and steroid responsive elements, allowing inducible and tissue-specific gene expression [7,8,9,10]; and (iv) encoding a unique post transcriptional regulatory system that can enhance gene expression [11,12,13,14,15].

Unlike the lentiviruses that are assembled at the plasma membrane, MMTV is a *Betaretrovirus* that displays a type B morphology [16] during replication where intracellular virus particles can be observed. Little is known about how the virus particle is assembled and in particular, the molecular mechanisms of MMTV genomic RNA (gRNA) packaging—a process that allows the virus to incorporate two copies of its single-stranded RNA genome into the assembling virus particle [17,18,19,20,21,22,23,24]. Retroviral RNA packaging requires specific interactions between both the gRNA and viral structural proteins, in particular Gag [25,26,27,28]. Like most retroviruses, an early study suggested that MMTV harbors sequences responsible for gRNA packaging at the 5′ end of its genome [29]. Recently, employing an in vivo packaging and transduction assay developed in our laboratory [30], we have shown that the 5′ untranslated region (5′ UTR) and the first 120 nucleotides (nts) of the *gag* gene are required for efficient MMTV gRNA packaging and propagation [31]. To establish the structural basis of MMTV gRNA packaging, these sequences were folded using minimum free energy algorithm programs like Mfold and RNAstructure [32,33]. The folding predictions of these sequences revealed a higher order structure comprising of several structural motifs, which could be involved during MMTV gRNA packaging [34]. Later, this structure was validated by SHAPE (selective 2′-hydroxyl acylation analyzed by primer extension), and the structure-function relationship of various structural motifs during MMTV gRNA packaging and dimerization was established [34]. Furthermore, it was established that there is a structural motif known as single-stranded purines (ssPurines) in the form of an apical loop which has been proposed to be the potential primary Gag binding site during the process of MMTV gRNA packaging [34]. 

Retroviral Gag polyproteins comprise of several domains that form the structural elements of the viral particle. Of these, the major domains are the matrix (MA), capsid (CA) and nucleocapsid (NC). The NC serves as the key player in selective gRNA packaging. It is a highly basic protein containing zinc finger motifs to facilitate protein-RNA interactions [17,35]. By conducting mutational analysis, it has been established that the Gag NC domain of number of retroviruses is the most vital protein domain involved in the gRNA packaging process [36,37,38,39,40]. However, other Gag domains may also be important for facilitating Gag-RNA interactions, such as the MA [41], CA [42], the p2 spacer peptide between CA and NC [43,44,45], and, in the case of HIV-1, the terminal p6 late domain [46]. Additionally, it has been suggested that NC probably recognizes dimeric genomes, since dimerization is a prerequisite for RNA packaging [47,48]. This interaction is thought to initiate a cascade of events that leads to the oligomerization/multimerization of the Gag polyprotein using gRNA as the substrate, which eventually leads to packaging of the gRNA into the newly forming virus particles. A number of studies have shown that specific selection of gRNA over the cellular and spliced RNAs is a multifaceted phenomenon that has been shown to occur in the context of the whole Gag polyprotein, especially in the case of HIV-1 [25,26,27,28]. Hence, it is not surprising that our limited understanding towards the highly selective packaging of gRNA by retroviral particles is predominantly due to the unavailability of biologically active full-length Gag polyprotein.

The MMTV Pr77^Gag^, encoded by the *gag* gene, is a precursor polypeptide, processed by the viral protease into its constituent domains NH2-p10 (MA), pp21, p3, p8, n, p27(CA), and p14(NC)-COOH [49,50]. Like most retroviruses, the MMTV Pr77^Gag^ assembles into an immature capsid and the proteolytic maturation takes place coupled with release from the cell [51,52]. The Pr77^Gag^ polyprotein plays a key role in selectively packaging the full length unspliced gRNA from a pool of cellular and spliced RNAs during viral assembly. The precise mechanism(s) by which Pr77^Gag^ accomplishes this specific selection is yet to be established. For example, it remains rather ambiguous whether the binding of Pr77^Gag^ to gRNA is based on an intrinsic capability of the polyprotein that allows specific selection of gRNA over its spliced variants, or whether other steps in the retroviral life cycle such as the nucleo-cytoplasmic transport and cellular compartmentalization of gRNA are also involved in discriminating gRNA during encapsidation process, as has been suggested for HIV-1 [53,54,55,56,57,58,59]. Thus, to delineate the molecular mechanism of MMTV gRNA packaging, it is critical to understand the biophysical and biochemical properties of full length Pr77^Gag^. However, the expression and purification of the biologically active MMTV full-length Pr77^Gag^ has not been accomplished in bacteria, although certain other MMTV proteins have been successfully purified after being expressed in bacteria, such as the *gag-pro* transframe protein p30 and reverse transcriptase [60,61]. 

In this study, we report the successful expression and purification of large amounts of full-length Pr77^Gag^ in soluble fractions of *Escherichia coli* (*E. coli*) containing a hexa-histidine (His_6_) tag at C-terminus. The purified MMTV Pr77^Gag^ could assemble in vitro into virus-like particles (VLPs), form intracellular VLPs in bacteria and eukaryotic cells, and most importantly, successfully package MMTV RNA. Thus, efficient expression and purification of full-length Pr77^Gag^ should allow us to investigate the differential binding ability of this protein to the unspliced full-length gRNA during selective RNA packaging process. This should additionally widen our understanding of the mechanisms of RNA–protein interaction(s) involved in gRNA packaging during MMTV life cycle.

## 2. Materials and Methods 

### 2.1. Nucleotide Numbers

All nucleotide numbers in this study refer to the MMTV genome pertaining to Genbank accession number AF228550.1 [62].

### 2.2. Full-Length Recombinant Gag Prokaryotic Expression Plasmids

**Figure 1:**
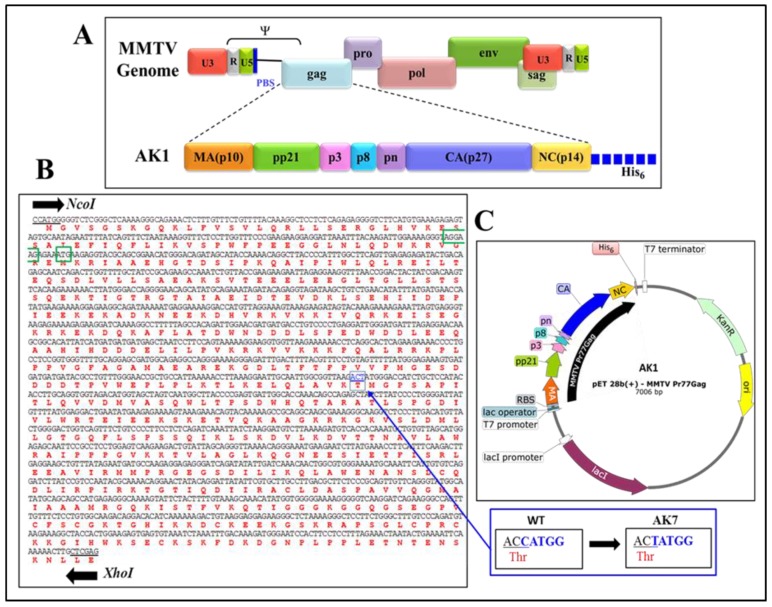
Construction of the recombinant full-length Pr77^Gag^ bacterial expression vector. (**A**) Domain organization of the mouse mammary tumor virus (MMTV) Gag precursor with His_6_-tag; (**B**) Nucleic acid and amino acid sequences of full-length MMTV *gag* gene. An internal *Nco*I site (boxed in blue color) was removed by introducing a silent mutation (shown in the inset) that preserved the threonine (Thr) amino acid. The Shine-Dalgarno-like sequence and second in-frame ATG are highlighted by green color; (**C**) Schematic representation of bacterial expression plasmid AK1 containing full-length MMTV Pr77^Gag^ gene cloned into the *Nco*I and *Xho*I sites of the pET28b(+) vector.

MMTV full-length *gag* gene (Pr77^Gag^; nucleotides 1485–3260) was commercially synthesized (Macrogen, South Korea) with flanking *Nco*I and *Xho*I sites and cloned into the bacterial expression vector pET28b(+) (Figure 1). Since the *gag* gene contain an inherent *Nco*I site at nt 2389, a silent mutation was created at this site by introducing a one nucleotide modification (*ACC*ATGG was changed to *ACT*ATGG, while maintaining the amino acid threonine, encoded by the italicized codon), resulting in only one *Nco*I site, to facilitate the cloning process. Using *Nco*I and *Xho*I sites during cloning resulted in placing the *gag* sequences in-frame with a hexa-histidine sequence that allowed the addition of a His_6_-tag at the C-terminus of recombinant Pr77^Gag^ protein with a predicted molecular weight of 65,890 Da, creating a molecular clone, AK1 (Figure 1). AK1 was further modified in such a fashion that a potential N-terminal-truncated protein from a second in-frame AUG, located at nts 1674–1676, is not expressed. Towards this end, a region harboring the Shine-Dalgarno-like sequence (underlined; 5′ AAAAGGGTAGGAAGAGAAATG 3′), located four nucleotides upstream of the second in-frame AUG [63] was silently mutated and the substituted nucleotides are underlined and shown in bold (5′ AA**GC**G**C**GT**G**GG**CC**G**C**GA**G**ATG 3′) without disrupting the amino acid sequence. These modifications generated the prokaryotic expression plasmid, AK7. AK7 was further modified by introducing a stop codon at the end of the *gag* sequence to create AK31 in a fashion that it expressed the full-length MMTV Pr77^Gag^ without the His_6_-tag. The resultant clones were sequenced to ensure that they were devoid of any mutations.

### 2.3. Full-Length Recombinant Gag Eukaryotic Expression Plasmids

The MMTV *gag* genes cloned into the prokaryotic expression vector (both with and without the His_6_-tag) was re-cloned into the eukaryotic expression vector, pCDNA3, using polymerase chain reaction (PCR). Towards this end, the same forward primer OTR1333 was used along with the reverse primer, OTR1335 that introduced the His_6_-tag at the end of *gag* gene using the template AK7. In order to introduce an *Xho*I restriction site (italicized) for cloning purposes and the Kozak sequences (underlined) at the 5′ end of the *gag* gene, an OTR1333 (5′ CCG *CTCGAG*GCCGCCACCATGGGGGTCTCGGGCTCAAAA 3′) forward primer was employed. Employing similar strategy for introducing the His_6_-tag (underlined) just upstream of the *gag* stop codon, followed by an *Xho*I endonuclease site (italicized) OTR1335 (5′ CCG*CTCGAG*TTA GTGGTGGTGGTGGTGGTGCAAGTTTTTTGA ATTTTCAGTATTAGTTTC 3′) was used. In order to create a eukaryotic Gag expression plasmid which did not contain the His_6_-tag but did contain an *Xho*I restriction site (italicized) immediately downstream of the stop codon, the reverse primer OTR1322 (5′ CCG*CTCGAG*TTA CAAGTTTTTTGA 3′) was used. PCR was performed as follows: Initial denaturation at 98 °C for 30 s, then 15 cycles of denaturation at 98 °C for 10 s, primer annealing at 62 °C for 30 s, followed by primer extension at 72 °C for 30 s and a final extension at 72 °C for 10 min. The amplified products were digested with *Xho*I endonuclease and cloned into pCDNA3 previously digested with the *Xho*I to create clones AK9 and AK10 (with and without His_6_-tag, respectively). Finally, to ensure proper nuclear export and translation of Gag mRNA, a PCR-amplified fragment containing the MPMV constitutive transport elements (CTE; [30,63]) with flanking *Xba*I sites was cloned into AK9 and AK10 previously digested with *Xba*I generating clones, AK13 and AK14. All clones were confirmed by sequencing. 

### 2.4. Escherichia coli Strains and Growth Media

The cloning of different expression plasmids was performed using the DH5α strain of *E. coli* using the conventional heat shock protocol with appropriate antibiotics (kanamycin; 50 µg/mL, ampicillin; 100 µg/mL). For prokaryotic protein expression, clones AK1, AK7, and AK31 were transformed into the BL21(DE3) strain of *E. coli*, cultured in Luria-Bertani (LB) medium (1% (*w*/*v*) peptone, 0.5% (*w*/*v*) yeast extract, and 0.5% NaCl) supplemented with kanamycin (50 µg/mL).

### 2.5. Expression of Recombinant Full-Length Pr77^Gag^-His_6_-Tagged Protein in Bacteria

For large scale expression of recombinant Pr77^Gag^-His_6_-tag protein, a single colony of transformed *E. coli* BL21(DE3) cells was inoculated into 50 mL of LB media containing kanamycin antibiotic (50 µg/mL), then cultured at 37 °C overnight with agitation at 200 rpm. The overnight culture was sub-cultured into 500 mL LB supplemented with the same concentration of kanamycin and 1% glucose in 2-L baffled flasks. The cultures were allowed to grow at 28 °C till an OD_600_ of 0.6 was achieved. Cultures were then induced with 0.4 mM of isopropyl β-d-1-thiogalactopyranoside (IPTG) and the cells were grown for an additional 4 h at 28 °C. Cells were pelleted by centrifugation at 4 °C for 15 min at 6300× *g* and stored at −80 °C until processed. 

### 2.6. Affinity Purification and Gel Filtration Chromatography

Purification of the recombinant Pr77^Gag^-His_6_-tag protein was carried out as previously described [46,64,65]. Frozen bacterial pellets were lysed in chilled CelLytic B buffer (Sigma-Aldrich, Saint Louis, MO, USA) supplemented with 5 U/mL Benzonase (Merck, Kenilworth, NJ, USA), 0.2 mg/mL of lysozyme (Sigma-Aldrich), and 1× ethylenediaminetetraacetic acid (EDTA) free protease inhibitor (Roche, Basel, Switzerland). The lysate was then centrifuged at 48,000× *g* for 1 h at 4 °C and the 4× binding buffer (0.2 M Tris-HCl of pH 8.0, 4.0 M NaCl, 40 mM β-mercaptoethanol, 10 mM dithiothreitol, 100 mM imidazole, 0.4% (*w*/*v*) Tween-20) was used to dilute the supernatant to a final concentration of 1×. Prior to loading onto 5 mL HisTRAP fast flow (FF) column (GE Healthcare, Little Chalfont, UK) which was pre-equilibrated with a buffer (with 50 mM Tris-HCl (pH 8.0), 1.0 M NaCl, 10 mM β-mercaptoethanol, 2.5 mM dithiothreitol, 25 mM imidazole, 0.1% (*w*/*v*) Tween-20, and 10% (*v*/*v*) glycerol), the lysate was filtered through a 0.4 µm polyethersulfone (PES) syringe filter. After loading the filtrate, the column was washed with the same buffer except for increasing the concentration of imidazole to 50 mM and the protein that bound to the column was eluted with buffer containing 250 mM imidazole.

The HisTRAP FF eluted Pr77Gag-His_6_-tag protein was concentrated using Amicon Ultra 15 column (with a 30 kDa cut-off) (Merck) for fractionation by gel filtration/size exclusion chromatography using a Superdex 200 increase 10/300 GL column (GE Healthcare) which was equilibrated with 50 mM Tris-HCl (pH 8.0) and 1.0 M NaCl. Peak fractions were analyzed using sodium dodecyl sulfate-polyacrylamide gel electrophoresis (SDS-PAGE) and fractions containing Pr77^Gag^-His_6_-tag protein were pooled and stored at −80 °C for downstream processing. The purity of the protein was established by measuring the absorbance ratio at 260 and 280 nm. 

### 2.7. Expression of Recombinant Full-Length Pr77^Gag^-His_6_-Tagged Protein in Eukaryotic Cells

The expression of Gag in eukaryotic cells was monitored in transient transfections using calcium phosphate kit (Invitrogen, Carlsbad, CA, USA) in HEK 293T cells. The transfections were carried out in triplicates in 6-well plates using 4 micrograms (µg) of full-length Gag eukaryotic expression plasmids (AK13 or AK14) along with 2 µg of MMTV-based transfer vector, DA024 [30]. To monitor transfection efficiencies, a secreted alkaline phosphatase expression plasmid (pSEAP, at a concentration of 100 ng per well) was also included in the transfections. Approximately 72 h post transfection, supernatants from the transfected cultures were harvested and subjected to low-speed centrifugation (3700× *g* for 10 min) to clear cellular debris. The clarified supernatants were then filtered through 0.2 µm cellulose acetate syringe filters, and subjected to ultracentrifugation at 70,000× *g* with a 20% (*w*/*v*) sucrose cushion to pellet the VLPs. The pelleted VLPs were then resuspended in TN buffer (20 mM Tris-HCl, pH 7.4, 150 mM NaCl) and subjected to RNA extraction (TRIzol) and western blotting. 

### 2.8. Estimation of RNA Packaging Potential by Reverse Transcriptase PCR

Packaging of MMTV RNA into the Gag-VLPs was tested by RT-PCR. Both cytoplasmic and viral RNA preparations were DNase-treated with TURBO DNase (Invitrogen) and amplified using transfer vector (DA024)-specific primers OTR671 (5′ GTCCTA ATATTCACGTCTCGTGTG 3′) and OTR672 (5′ CTGTTCGGGCGCCAGCTGCCGCAG 3′) to confirm the successful removal of contaminating plasmid DNA in the extracted RNA preparations. The cDNA synthesis from the DNased-RNAs was accomplished using random hexamers (5′ NNNNNN 3′) and MMLV reverse transcriptase (Promega, Madison, WI, USA) as previously described [66,67]. To monitor the ability of Pr77^Gag^ VLPs to package transfer vector (DA024) RNA, complementary DNAs (cDNAs) were amplified using the same vector-specific primers (OTR671 and OTR672). 

### 2.9. Sodium Dodecyl Sulfate-Polyacrylamide Gel Electrophoresis and Western Blotting

SDS-PAGE and western blotting were used to monitor the expression and purification of recombinant Pr77^Gag^-His_6_-tag protein. Protein samples were analyzed on 4–12% ExpressPlus^TM^ PAGE gel (GenScript, Piscataway, NJ, USA), electrophoresed under reducing conditions using 3-(*N*-morpholino)propanesulfonic acid (MOPS) buffer (GenScript), and stained with Coomassie Brilliant Blue. For western blot analyses, duplicate gels were transferred onto nitrocellulose membranes and probed with α-MMTV p27 CA monoclonal antibody Blue 7 [68] and an α-His_6_ monoclonal antibody-horseradish peroxidase (HRP) conjugate (Sigma-Aldrich). 

### 2.10. Detection of Prokaryotically-Expressed Virus-Like Particles Using Transmission Electron Microscopy

To observe VLPs formed by recombinant Pr77^Gag^-His_6_-tag protein in bacterial cells (following IPTG induction), the pelleted cells were washed with 0.1 M phosphate buffered saline (PBS) and fixed in Karnovsky’s fixative overnight. Cell pellets were then stained with 1% osmium tetroxide and subjected to graded ethanol dehydration. The pellets were then embedded in epoxy resin (Agar 100). Ultrathin (95 nm) sections of the embedded samples were fixed on 200 mesh copper (Cu) grids and negatively stained with 1% uranyl acetate and lead citrate as a double stain. The sections were analyzed using a FEI Tecnai Biotwin Spirit G2 transmission electron microscope. 

### 2.11. In Vitro Assembly of Virus-Like Particles from Bacterially-Expressed Recombinant Full-Length Pr77^Gag^-His_6_-Tag Protein

The purified recombinant Pr77^Gag^-His_6_-tag protein expressed in bacteria was resuspended in assembly buffer (50 mM Tris (pH 7.4), 1.0 M NaCl) at a concentration of 2 mg/mL and incubated with yeast tRNA at a nucleic acid to protein ratio of 4% (*w*/*w*). This mix was placed in a Slide-A-Lyzer 10K dialysis cassette G2 (Thermo Scientific, Waltham, MA, USA) and dialyzed against dialysis buffer (20 mM Tris (pH 7.4), 150 mM NaCl and 10 mM dithiothreitol) overnight at 4 °C. Following overnight dialysis, ~8–10 µL of the dialyzed solution was spotted onto a carbon-coated formvar grid (Proscitech, Kirwan, Australia), air dried, and stained with 1% uranyl acetate for observation using transmission electron microscopy (TEM).

## 3. Results and Discussion

### 3.1. Successful Expression of Full-Length Recombinant MMTV Pr77^Gag^-His_6_-Tagged Protein in Bacteria

For the expression of full-length MMTV Pr77^Gag^ which contained a C-terminus His_6_-tag, a recombinant bacterial expression plasmid (AK1; Figure 1) was generated. High level expression of His-tagged MMTV Pr77^Gag^ with a predicted molecular weight of ~65,890 Da in BL21(DE3) bacterial cells was achieved by induction with IPTG (Figure 1).

**Figure 2:**
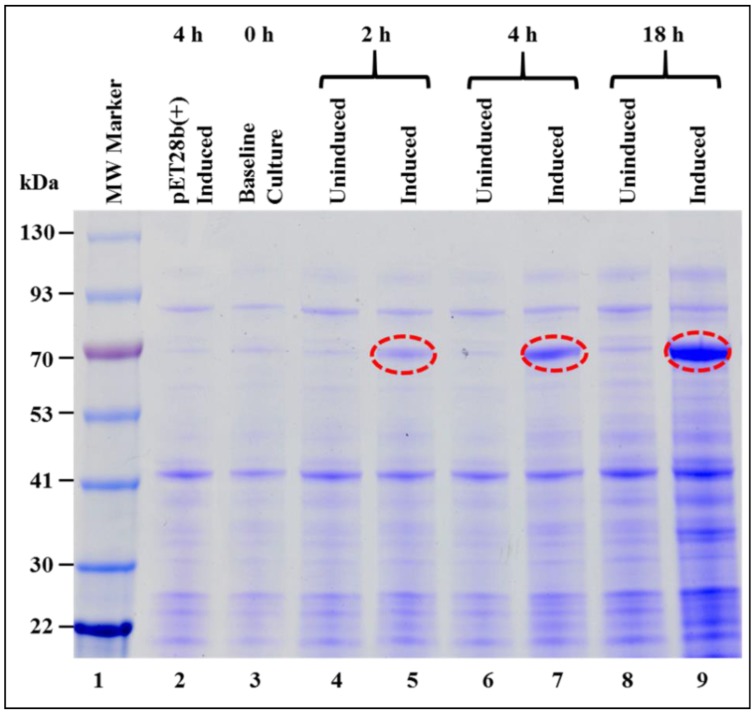
Expression of recombinant Pr77^Gag^-His_6_-tag fusion protein in *Escherichia coli* (*E. coli*). Sodium dodecyl sulfate-polyacrylamide gel electrophoresis (SDS-PAGE) analysis showing full length Pr77^Gag^-His_6_-tag fusion protein expressed from AK1 in total bacterial cell lysates at 0, 2, 4, and 18 h post IPTG-induction and un-induced BL21(DE3) bacterial cells. The bacterial cells were grown at 37 °C overnight, but following IPTG induction, cultures were grown sub-optimally at 28 °C. MW: molecular weight.

The expression of the recombinant Pr77^Gag^-His_6_-tagged protein in total bacterial lysates was monitored at 0, 2, 4, and 18 h post induction, by SDS-PAGE (Figure 2). Bands corresponding to the size of recombinant Pr77^Gag^-His_6_-tagged protein (70 kDa) were observed only in the IPTG induced cultures at 2, 4, and 18 h (Figure 2, lanes 5, 7, and 9) but not in cultures at 0 h (lane 3) or un-induced cultures at 2, 4, and 18 h (Figure 2, lanes 4, 6, and 8), as well in cultures transformed with only pET28b(+) expression vector (Figure 2, lane 2). This is despite the presence of the “poison sequences” present in Gag that are presumably “toxic” for bacteria [69]. It is possible that we did not see the effect of these poison sequences due to the inducible and suboptimal nature of our expression system, allowing the bacteria to survive for short periods under these conditions. 

### 3.2. Full-Length MMTV Pr77^Gag^-His_6_-Tagged Fusion Protein Is Expressed in the Soluble Form in Bacteria

Next, we wanted to establish whether the recombinant MMTV Pr77^Gag^-His_6_-tagged fusion protein was expressed in the soluble bacterial fraction so that it could be purified. Towards this end, the large-scale expression of recombinant MMTV Pr77^Gag^ was performed at sub-optimal conditions such as low temperature (28 °C) and shorter duration (4 h only) as described in Materials and Methods. This was based on the earlier observations that in case of Mason-Pfizer monkey virus (MPMV) Pr78^Gag^, culturing bacteria at 37 °C post-induction resulted in the confinement of MPMV Gag polyprotein in the inclusion bodies containing aberrantly assembled spiral like structures [70]. Removal of insoluble material (cell debris and/or inclusion bodies) was accomplished by centrifugation of the bacterial lysates. The soluble fractions from different cultures were analyzed for the expression of full-length MMTV Pr77^Gag^-His_6_-tag fusion protein.

**Figure 3:**
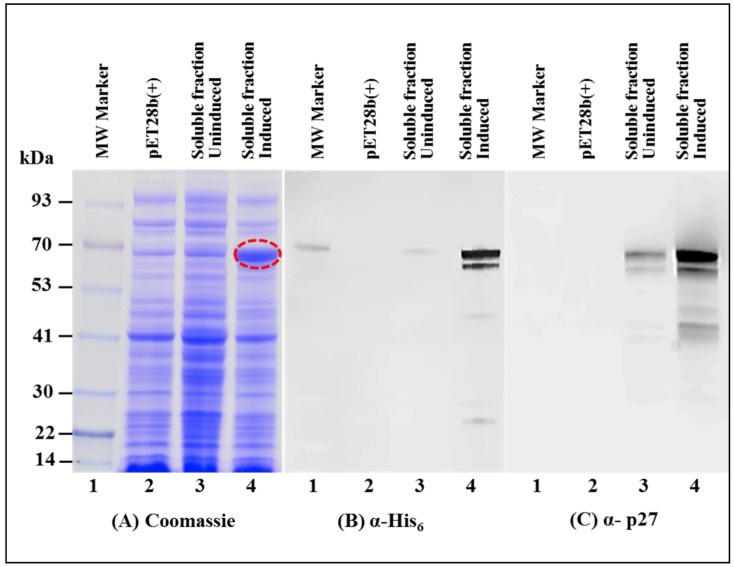
Recombinant Pr77^Gag^-His_6_-tag fusion protein expressed in soluble fraction of *E. coli*. (**A**) SDS-PAGE analysis showing recombinant MMTV Pr77^Gag^-His_6_-tag fusion protein expression in the bacterial soluble fraction (lane 4) transformed with AK1; (**B**) western blot analysis of MMTV Pr77^Gag^ expression by AK1 in soluble fraction analyzed with an α-His_6_ monoclonal antibody (lane 4); and (**C**) with an α-p27 monoclonal antibody (lane 4), respectively.

As expected, SDS-PAGE analysis of the soluble fraction revealed a very distinctive band of ~70 kDa which corresponded to the size of recombinant MMTV Pr77^Gag^-His_6_-tag fusion protein (Figure 3A; lane 4). Immunoblotting on AK1 un-induced culture lysates using α-His_6_ monoclonal antibody as well as α-MMTV p27 monoclonal antibody also showed low level expression of MMTV Pr77^Gag^-His_6_-tag fusion protein (Figure 3B,C; lane 3). Such low level expression in the un-induced culture could possibly be due to the leaky nature of the bacterial promoter (discussed later). 

**Figure 4:**
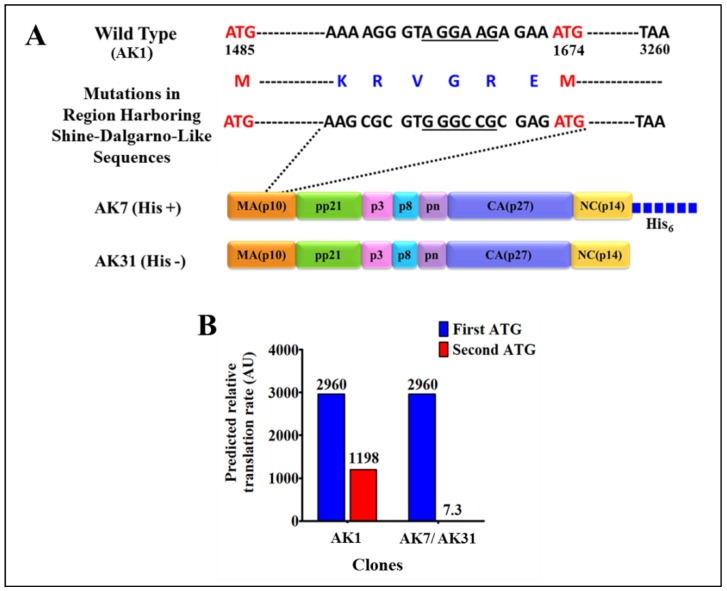
Silent mutations in the Shine-Dalgarno-like sequence and the predicted relative translation rates from the first and the second in-frame ATGs. (**A**) Illustration of the 18-nucleotide region mutated in MMTV *gag* gene to disrupt the Shine-Dalgarno-like sequence (underlined) 4 nts upstream of the second in-frame ATG (at nucleotide position 1674). These mutated sequences were cloned in both with and without His-tag clones AK7 and AK31, respectively; (**B**) Bar graphs showing the predicted translation rates from the legitimate first start codon and the second in-frame start codon in the wild type and in AK7(His+) and AK31(His−)-containing a mutated Shine-Dalgarno-like sequence.

Immunoblotting on IPTG induced soluble fractions using HRP-conjugated α-His_6_ monoclonal antibody (Figure 3B; lane 4) as well as α-MMTV p27 monoclonal antibody (Figure 3C; lane 4) confirmed the identity of the ~70 kDa band; however, another band of a slightly lower molecular weight was also observed. Careful analysis of the MMTV full-length gag sequence suggested that the second band could be due to the expression of a truncated protein from a second in-frame AUG (nts 1674–1676) located 189 nts downstream from the canonical AUG (nts 1485; Figure 4A). Expression of the truncated Gag protein from this internal AUG was possibly facilitated by the presence of a Shine-Dalgarno-like sequence (AGGAAG; Figure 4A), located 4 nts upstream of the in-frame second AUG [71]. This was confirmed by calculating the relative translation rates from both the first AUG as well as from the second in-frame AUG employing an online program called RBS Calculator v2.0 [72,73]. Relative translational rate analysis revealed that the predicted translation rate from the first AUG was 2960 arbitrary units (au), whereas that from the second in-frame AUG was 1198 au (Figure 4B). Such a predicted translation rate corroborated well with the level of intensities of the bands following immunoblotting with the truncated protein being expressed approximately 1/3rd of the level of the full-length Gag (Figure 3B,C; lane 4). To eliminate expression from the second AUG, we introduced silent mutations in the 18 nts region (1656–1673) that included the Shine-Dalgarno-like sequence (AGGAAG), resulting in its disruption (from AGGAAG to GGGCCG), but without changing the amino acids sequence (Figure 4A). These changes reduced the predicted translation rate from the second in-frame AUG to almost negligible levels (from 1198 to 7.3 au; Figure 4B). 

**Figure 5:**
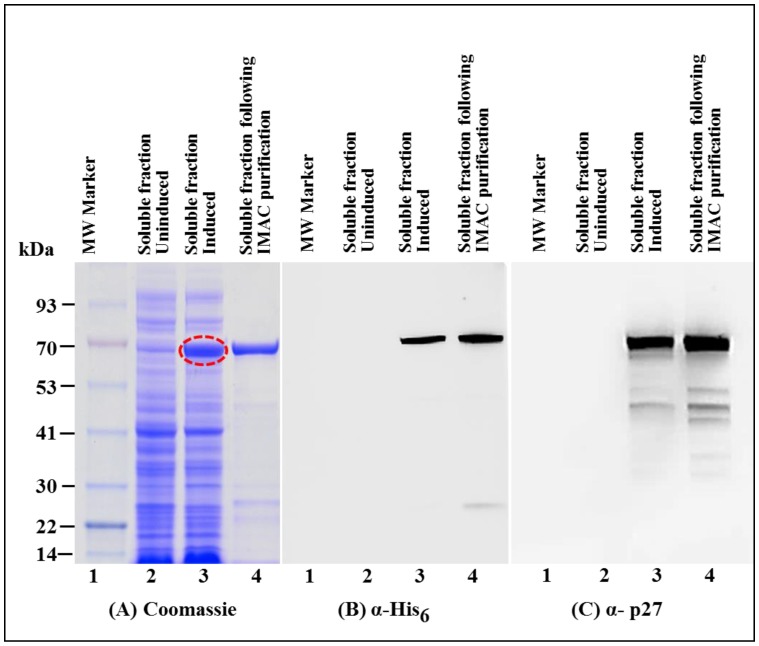
Expression of recombinant Pr77^Gag^-His_6_-tag fusion protein from AK7 in soluble fractions of *E. coli* before and after immobilized metal affinity chromatography (IMAC) purification. (**A**) SDS-PAGE analysis showing recombinant MMTV Pr77^Gag^-His_6_-tag fusion protein expressed in the bacterial soluble fractions transformed with AK7 (lane 3), followed by IMAC purification (lane 4); (**B**) western blot analysis of MMTV Pr77^Gag^-His_6_-tag fusion protein analyzed with an α-His_6_ monoclonal antibody; and (**C**) with α-p27 monoclonal antibody, respectively.

Next, the modified full-length *gag* gene was cloned into pET28b(+) bacterial expression vector to generate the final clone, AK7 that was tested further. Since earlier we had observed that the pET T7 promoter may be leaky (Figure 3B,C; lane 3), AK7 was cultured in the presence of glucose, as described previously to inhibit promoter leakiness [74]. As expected, results shown in Figure 5 further confirm that the recombinant Pr77^Gag^-His_6_-tagged fusion protein was observed only in the induced culture and not in the un-induced culture (Figure 5A–C; lane 2). Consistent with the predicted relative translation rates, immunoblotting of bacterial soluble fractions from AK7 using α-His_6_ monoclonal antibody as well as α-MMTV p27 monoclonal antibody revealed that mutation of the Shine-Dalgarno-like sequence was sufficient to disable the expression of the truncated Pr77^Gag^-His_6_-tag fusion protein (Figure 5B; lane 3 and Figure 5C; lane 3). Finally, in addition to the desired Pr77^Gag^-His_6_-tag fusion protein, some spurious bands were also observed in immunoblots using α-MMTV p27 monoclonal antibody (Figure 5C; lane 3). These nonspecific bands could be due to the possible degradation of our recombinant protein and could be removed following size exclusion chromatography (described later). Taken together, these results clearly demonstrate that the MMTV recombinant Pr77^Gag^-His_6_-tag protein could be expressed primarily in the soluble fraction of bacterial lysates. 

### 3.3. Immobilized Metal Affinity Chromatography Purification of the Soluble Fraction Containing Recombinant Full-Length Pr77^Gag^-His_6_-Tagged Fusion Protein

After establishing that the expressed recombinant Pr77^Gag^-His_6_-tag protein was present in the soluble fraction, purification of the recombinant Pr77^Gag^-His_6_-tag protein from the bacterial lysate was performed by employing immobilized metal affinity chromatography (IMAC), as described in Materials and Methods. The non-denaturing buffering conditions (especially the presence of 1.0 M NaCl) were used to facilitate the binding of the protein to the column and to circumvent protein aggregation and precipitation. Following IMAC purification the purity of the recombinant MMTV Pr77^Gag^-His_6_-tag protein was established by SDS-PAGE and immunoblotting. Coomassie Brilliant Blue staining of the gels revealed that the bacterial proteins that were largely present in the soluble fraction before IMAC purification were removed during IMAC purification (Figure 5A; compare lane 3 with lane 4). Immunoblotting of IMAC-purified protein with α-His_6_ (Figure 5B; lane 4) and α-MMTV p27 monoclonal antibodies (Figure 5C; lane 4) still showed several additional bands, possibly due to degradation of the fusion protein (Figure 5C) which were successfully removed following size exclusion chromatography (described later). These results confirm that the presence of His_6_-tag at the C-terminus of MMTV full-length Gag not only allowed its binding to the HisTRAP column but also facilitated elution in a much purer form (Figure 5A; compare lane 3 with lane 4).

### 3.4. Gel Filtration Chromatography Purification of the IMAC-Purified Recombinant Full-Length Pr77^Gag^-His_6_-Tagged Fusion Protein

**Figure 6:**
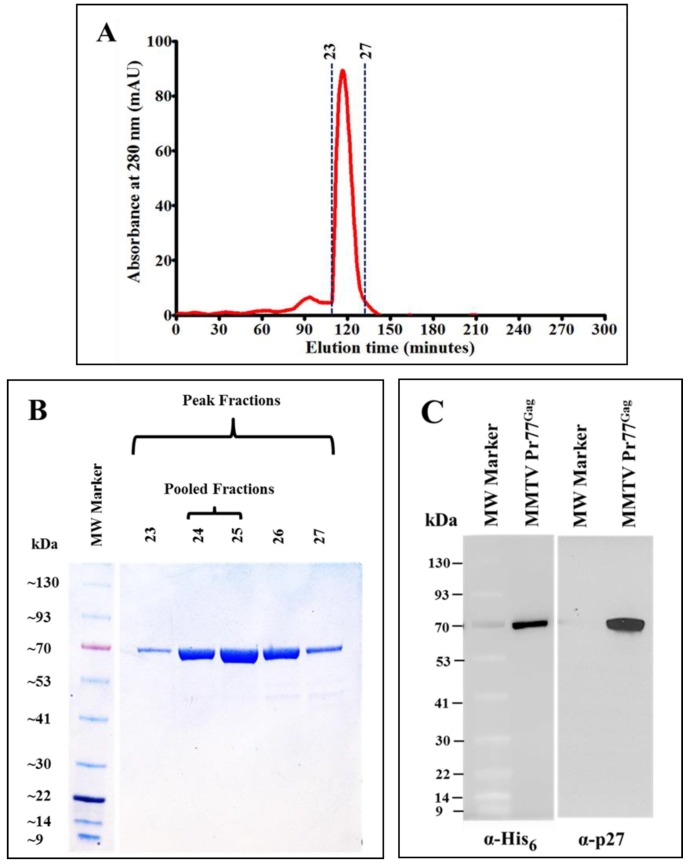
Resolution of IMAC-purified recombinant Pr77^Gag^-His_6_-tag fusion protein by size exclusion chromatography and western blot analysis. (**A**) Absorbance versus elution time chromatogram of eluted fractions after size exclusion chromatography; (**B**) Coomassie Brilliant Blue-stained SDS-PAGE analysis of peak fractions 23 to 27, showing the resolution of purified recombinant MMTV Pr77^Gag^ expressed from AK7; (**C**) western blot analysis of pooled peak fractions of purified MMTV Pr77^Gag^-His_6_-tag fusion protein analyzed with α-His_6_ and α-p27 monoclonal antibodies, respectively.

The protein eluted after IMAC purification was concentrated using an Amicon Ultra 15 centrifugal columns (30 kDa cut-off membrane). Further purification of the protein was carried out by size exclusion chromatography under non-denaturing conditions using a Superdex 200 10/300 GL column. The high salt concentration in the gel filtration buffer (1.0 M NaCl) prevented any possible protein aggregation and precipitation. 500 µL fractions were collected for several hours (Figure 6A) and protein fractions showing strong absorbance at 280 nm (fractions 23–27) were subjected to SDS-PAGE and western blot analyses. 

As shown in Figure 6B, fractions corresponding to the sharp peak consisted of pure MMTV Pr77^Gag^-His_6_-tag fusion protein with varying amounts of protein. Since fractions 26 and 27 showed an additional band of a smaller size, only fractions representing the highest amount of pure protein (peaks 24 and 25; Figure 6B) were pooled, concentrated and further analyzed by immunoblotting using α-MMTV p27 and α-His_6_ monoclonal antibodies. Figure 6C, in close corroboration with the observed SDS-PAGE analysis (Figure 6B), demonstrated that the pooled protein fractions contained pure MMTV Pr77^Gag^-His_6_-tag fusion protein. The purity of the protein was assessed to be greater than 95% as measured by the A_260_/A_280_ ratio by spectrophotometry (giving a value of 0.6), confirming that the purified protein contains only an insignificant level of nucleic acid contamination. From one liter of bacterial culture, ~4.4 mg protein was obtained after IMAC purification. When IMAC purified protein (4.4 mg) was subjected to gel filtration/size exclusion chromatography ~1.4 mg purified protein was recovered. 

### 3.5. In Vitro Assembly to Form Virus-Like Particles by the Recombinant Full-Length Pr77^Gag^-His_6_-Tagged Fusion Protein

The ability of a number of retroviral recombinant full-length Gag proteins to assemble in vitro to form VLPs have already been established [46,65,75,76]. Thus, we analyzed the in vitro assembling ability of our purified recombinant MMTV Pr77^Gag^-His_6_-tag fusion protein expressed from AK7. The in vitro assembly experiment was carried out in the presence of yeast tRNA since the presence of nucleic acids along with purified Gag protein has been shown to be a prerequisite for in vitro assembly of VLPs [46,65,75,76]. The protein-RNA mixture in a higher salt concentration buffer (1 M NaCl) was then subjected to dialysis against a buffer with physiological salt concentration. A sample with only yeast tRNA was also dialyzed in the same manner as a control. After overnight dialysis, the protein-RNA mixture was recovered from the dialysis cassette and 10 µL (~1/40th of the suspension) was spotted on a formvar carbon coated grids, and processed for TEM.

**Figure 7:**
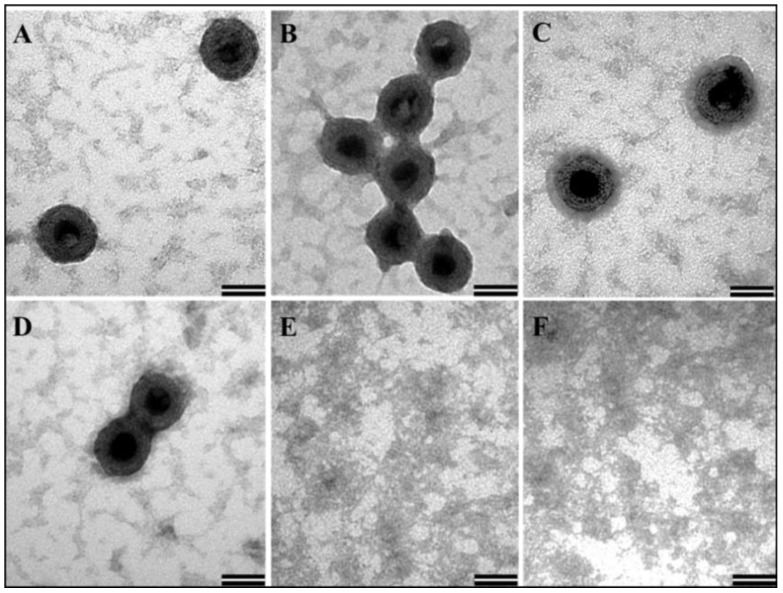
Transmission electron micrographs showing virus-like particles (VLPs) following in vitro assembly. (**A**–**D**) In vitro assembled VLPs from purified recombinant Pr77^Gag^-His_6_-tag fusion expressed from AK7 in the presence of yeast tRNA; and (**E**,**F**) negative controls consisting of assembly buffer and yeast tRNA in the absence of any protein (Scale bar = 50 nm, 135,000× magnification).

VLPs of approximately 62–66nm in size resembling immature virus particles were observed in various electron micrographs taken from different fields (Figure 7A–D). In contrast, as expected, yeast tRNA alone without any purified MMTV full-length Gag did not show any VLP-like structure (Figure 7E,F). Earlier studies have reported a smaller size VLPs (~20–30 nm) obtained following in vitro assembly using purified full-length Gag from HIV-1 and feline immunodeficiency virus (FIV; [46,65,75,76]) in contrast to the larger size of full-length Gag particles that have been observed in vivo in eukaryotic cells. However, in our case, the size of in vitro assembled VLPs are comparable to those observed in E. coli (discussed below) and suggest a unique property of MMTV, making this virus particularly interesting for in vitro assembly studies.

Prior to in vitro assembly, the protein was frozen and then thawed. The efficient formation of VLPs suggests that the biophysical activity of purified protein remained intact following a freeze-thaw cycle. From these experiments, it is clear that the purified MMTV recombinant full-length Gag-His_6_-tag fusion protein maintained its multimerizing/oligomerizing ability and assembled in vitro to form VLPs as reported previously in the case of HIV-1 and FIV [46,65,75,76].

### 3.6. Recombinant Full-Length MMTV Pr77^Gag^-His_6_-Tagged Protein Expressed in Bacteria Can Form Virus-Like Particles

**Figure 8:**
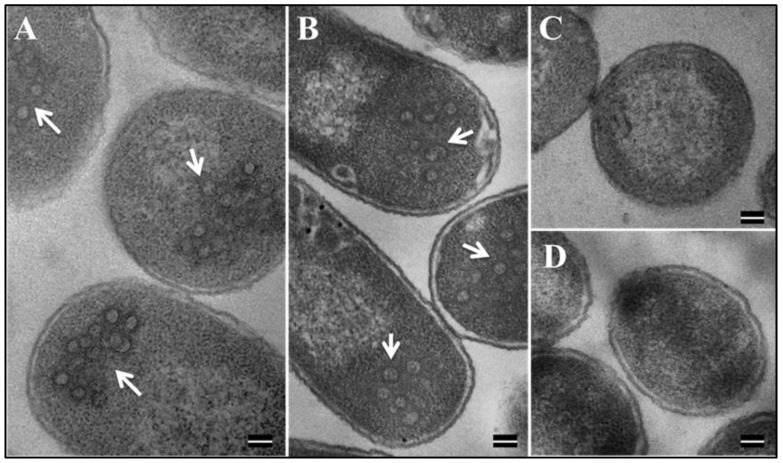
Formation of VLPs by recombinant Pr77^Gag^-His_6_-tag fusion in *E. coli* BL21(DE3). Transmission electron micrographs showing VLPs assembled from (**A**) recombinant Pr77^Gag^-His_6_-tag fusion expressed in *E. coli* BL21(DE3) cells transformed with AK7 and (**B**) AK31 (without His_6_ tag); (**C**,**D**) un-induced BL21(DE3) cells transformed with AK7 and AK31, respectively (Scale bar = 100 nm; 60,000× magnification).

The formation of VLPs by several retroviral Gag proteins expressed in bacteria has already been established [70,77,78,79,80]. Therefore, the ability of our recombinant MMTV Pr77^Gag^ proteins (both with and without the His_6_-tag), to assemble into VLPs was analyzed. TEM was performed on bacterial samples transformed with full-length MMTV Gag recombinant clone AK7 (with His_6_-tag) and AK31 (without His-tag) and cultured at 28°C post-IPTG induction. Ultrathin sections (95 nm) of the bacterial pellets were negatively stained with 1% uranyl acetate followed by lead citrate and visualized on TEM. Electron micrographs of E. coli BL21(DE3) cells transformed with both AK7 and AK31 showed intra-cytoplasmic electron dense rings of ~55–70 nm in size, closely resembling immature VLPs (Figure 8A,B). As expected, no VLPs were observed in un-induced bacterial cells that were transformed with either the AK7- or in AK31 expression plasmids (Figure 8C,D). Similarly, no such VLP structures were observed when the cloning vector by itself was transformed into BL21 (DE3) cells and induced with IPTG (data not shown). These results suggest that when expressed in bacteria, full-length MMTV Gag proteins either with or without the His_6_-tag were capable of forming morphologically indistinguishable VLPs (Figure 8A,B). 

### 3.7. Eukaryotically-Expressed, Full-Length Recombinant Pr77Gag His_6_-Tagged Fusion Protein Can Form Virus-Like Particles Competent to Package Unspliced Sub-Genomic RNA

**Figure 9:**
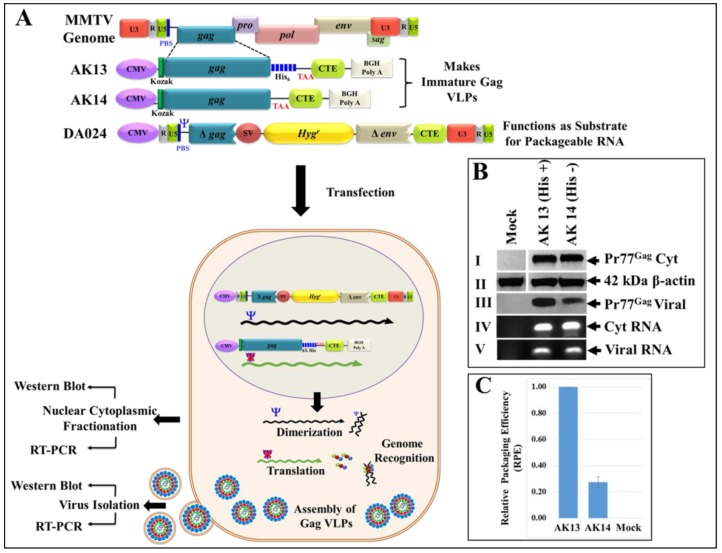
Schematic representation of the two-plasmid genetic complementation assay to demonstrate VLPs formation following Pr77^Gag^-His_6_-tag protein expression in eukaryotic cells and their ability to package MMTV sub-genomic RNA. (**A**) Upper panel; MMTV full-length Gag eukaryotic expression plasmids and MMTV sub-genomic transfer vector, DA024 [29]. (**A**) Lower panel; Graphical representation of the MMTV two-plasmid genetic complementation assay in which VLPs produced by recombinant MMTV Pr77^Gag^ expression plasmids (AK13 and AK14) should package MMTV sub-genomic transfer vector (DA024) owing to the presence of the packaging sequences (Ψ). HEK 293T cells co-transfected with the two plasmids were subjected to nucleocytoplasmic fractionation. The cytoplasmic fractions and pelleted VLPs were analyzed for transfer vector RNA expression by RT-PCR; (**B**) western blots performed on cell lysates and ultracentrifuged transfected culture supernatants using α-MMTV p27 monoclonal antibody (panels I and III), and α-β-actin antibody (panel II), respectively. PCR amplification of cDNAs prepared from cytoplasmic (panel IV) and viral RNA (panel V) demonstrating RNA packaging using MMTV transfer vector (DA024)-specific primers (OTR671/OTR672) to amplify a 142 bp fragment. The RNA packaging experiment was performed more than three independent times followed by its analysis by RT-PCR and a representative blot of the packaged viral RNA is shown in panel V; (**C**) relative RNA packaging efficiency (RPE) by AK13 and AK14 of one of the representative experiments, as measured by quantitative real time PCR. Briefly, the real time experiments were conducted in triplicates (± standard deviation (SD)) and the relative quantification (RQ) values obtained for the packaged viral RNA in the Gag VLPs were normalized to the cytoplasmic expression of the transfer vector RNA (DA024) for the respective clones as described previously.

Finally, we determined the in vivo expression and RNA packaging potential of MMTV Gag recombinant proteins in eukaryotic cells. Towards this end, both the His(+) and His(−) versions of the full-length MMTV gag gene were cloned into eukaryotic expression plasmid pCDNA3, creating AK13 (with His_6_-tag) and AK14 (without the His_6_-tag) (Figure 9A; upper panel). To ensure appropriate nuclear export and translation of the MMTV Pr77^Gag^ mRNA, a 231-nucleotide long MPMV CTE was cloned downstream of the MMTV Gag stop codon in both of these clones (Figure 9A upper panel). The MPMV CTE has previously been shown to be required for the successful expression of the MMTV *gag/pol* genes from eukaryotic expression vectors in the absence of a functional MMTV Rem/RmRE transport system [30]. These full-length Gag expression plasmids were tested for their ability to package MMTV sub-genomic RNA expressed from the transfer vector DA024 by co-transfection into the highly efficient HEK 293T cells, as described before [30].

Western blot analysis of cell lysates revealed successful expression of recombinant MMTV Pr77^Gag^ protein by both the His(+) and His(−) plasmids, AK13 and AK14, respectively (Figure 9B; panel I). These expressed proteins could form VLPs, as detected by their presence in the supernatants pelleted via ultracentrifugation followed by immunoblotting with α-MMTV p27 monoclonal antibody (Figure 9B; panel III). These data reveal that the inclusion of His_6_-tag at the C-terminus of MMTV full-length Pr77^Gag^ did not impinge upon the expression of recombinant full-length MMTV Pr77^Gag^, resulting in the formation of Gag VLPs in the eukaryotic cells. 

To establish whether Gag VLPs formed by the recombinant MMTV full-length Pr77^Gag^ can package MMTV sub-genomic RNA, RNAs from the cytoplasmic fractions of HEK293T cells as well as from the pelleted viral particles were extracted. The RNA preparations were DNase-treated to remove any contaminating plasmid DNA that may have been carried over from the transfected cultures. After confirming the absence of plasmid DNA by PCR using transfer vector RNA-specific primers (OTR671 and OTR672; data not shown), the DNased-RNAs were converted into cDNAs. Furthermore, the integrity of nucleocytoplasmic fractionation was ensured by testing for the absence of unspliced β-actin mRNA in the cytoplasmic fractions by RT-PCR, as described previously ([30]; data not shown)). Next, expression of the transfer vector (DA024) RNA was analyzed in the cytoplasmic fractions by RT-PCR, which ensured that these RNAs were stably expressed and exported to the cytoplasm so that they can function as competent substrates for RNA packaging in the assembling virus particles (Figure 9B; panel IV). 

Finally, the ability of the VLPs formed by the recombinant MMTV Pr77^Gag^-His_6_-tag fusion protein (AK13) as well as without His-tag (AK14) to package the DA024 transfer vector RNA was analyzed by preparing cDNAs from the RNA isolated from the pelleted VLPs. Finally, the relative packaging efficiency of the VLPs was assessed by employing the custom-designed Taqman gene expression assay previously used by our group to quantitate MMTV RNA packaging [31,34]. Results shown in Figure 9C confirm that both His(+) and His(−) Gag VLPs could successfully package MMTV sub-genomic RNA into the virus particles and in proportion to the amount of corresponding Gag VLPs (Figure 9B; panel III). These results suggest that the recombinant MMTV Pr77^Gag^-His_6_-tag fusion protein expressed in eukaryotic cells is biologically active, resulting in the formation of VLPs with the capability of encapsidating sub-genomic MMTV RNA. 

A point to note, we made a consistent observation that the amount of Gag VLPs from the His(−) vector AK14 was always less compared to the His(+) expression vector, AK13 (Figure 9B; panel III) despite their efficient expression in the cytoplasm (Figure 9B; panels I and II). This was true even when the experiments were repeated multiple times with different preparations of plasmid DNAs and having comparable transfection efficiencies from three independent experiments (SEAP relative luminescence units 1811946 for AK13 versus 1820756 for AK14). The exact reason for this differential Gag VLP formation in our experiments remains largely unclear. Irrespective of the amount of VLPs produced by the His(+) or His(−) expression vectors, they could package MMTV sub-genomic RNA efficiently and correspondingly to the amount of Gag VLPs formed (Figure 9B; panel III & Figure 9C). Thus, from a biological and functional perspective, both proteins seemed to have similar capabilities. Interestingly, when sequences for AK13 (with His_6_-tag) and AK14 (without His_6_-tag) were analyzed by ExPASy-Compute pI/Mw tool to calculate the theoretical isoelectric point (pI), it predicted a negligible effect (0.18) in these proteins (AK13 with His_6_-tag: pI: 6.76 versus AK14 without His_6_-tag: pI: 6.58), corroborating similarities in their functional observed capacities. 

## 4. Conclusions

Work presented in this study reports the successful cloning and expression of the recombinant full-length Pr77^Gag^ protein of MMTV both with and without a His_6_-tag. The protein could be expressed and purified from soluble fractions of bacteria at high levels, had the ability to form VLPs in vitro, and could also form VLPs in bacterial cells in vivo. VLPs formed by the recombinant full-length Gag protein in eukaryotic cells revealed their ability to recognize and encapsidate MMTV sub-genomic RNA successfully, despite the presence of His_6_-tag at the C-terminus. The availability of pure forms of MMTV Pr77^Gag^ should facilitate structural studies and further biochemical and functional characterization to better understand the molecular interactions that take place during RNA dimerization, packaging, and virus assembly steps critical for not only understanding virus replication, but also importantly for the development of MMTV-based vectors for human gene therapy.

## Supplementary Materials

Supplementary File 1
