# Supplementary material for: Biochemical and Functional Characterization of Mouse Mammary Tumor Virus Full-Length Pr77Gag Expressed in Prokaryotic and Eukaryotic Cells
Source: Viruses. 2018 Jun 18;10(6):334. doi: 10.3390/v10060334 (PMC6024702; doi:10.3390/v10060334)
Supplement: Supplementary File 1 [file viruses-10-00334-s001.pdf]

Supplementary Data

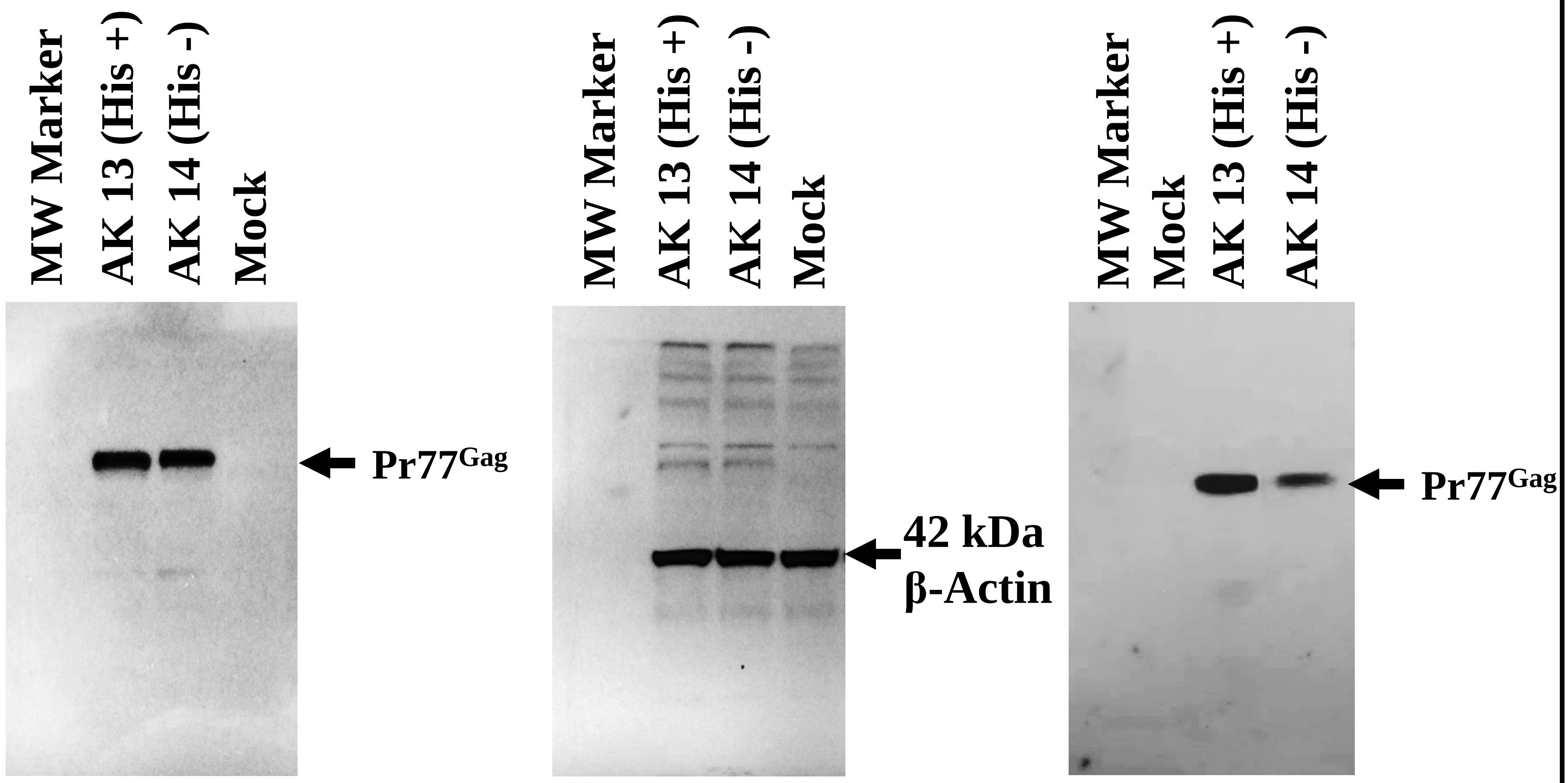

Cytoplasmic
Pr77[Gag]
Blot used for Figure 9B Panel I

Cytoplasmic
β-Actin
Blot used for Figure 9B Panel II

VLP
Pr77[Gag]
Blot used for Figure 9B Panel III

Supplementary Data

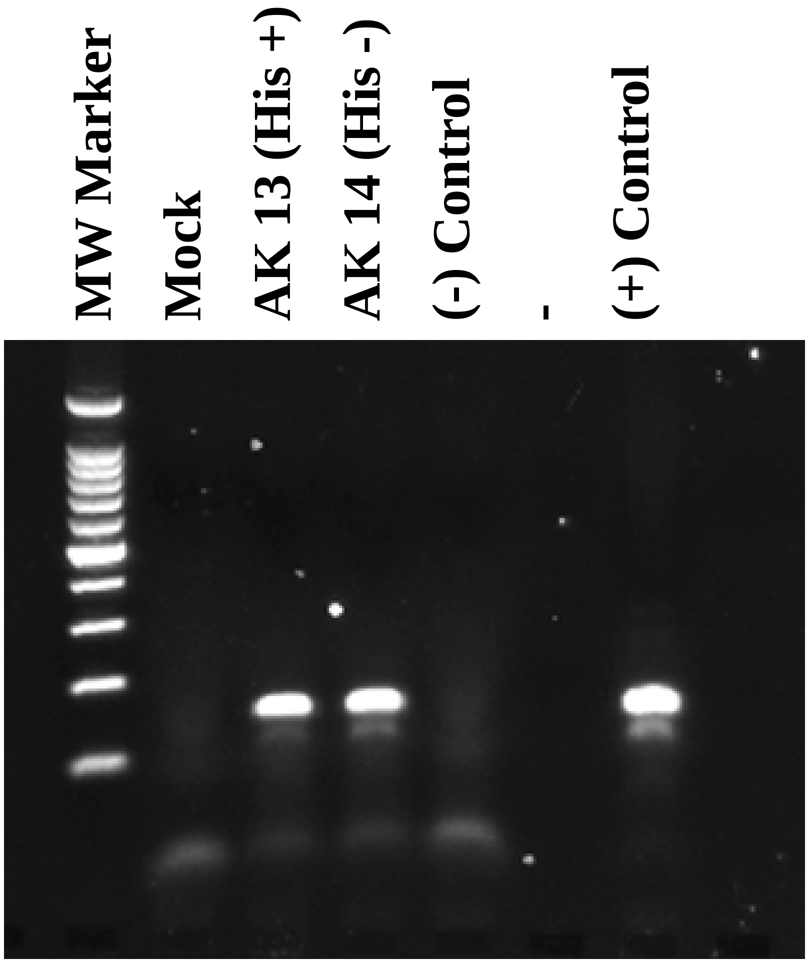

cDNAs from cytoplasmic RNA
Agarose gel picture used for Figure 9B Panel IV

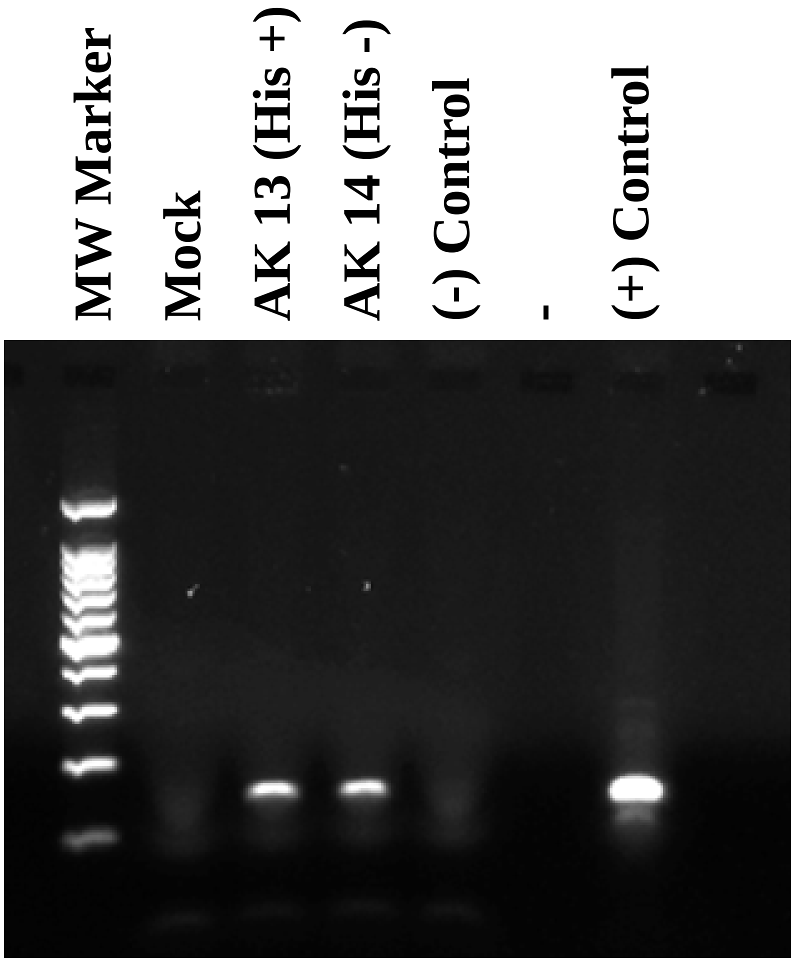

cDNAs from packaged viral RNA
Agarose gel picture used for Figure 9B Panel V
